# One-Year Mortality in Patients Undergoing Transcatheter Aortic Valve Replacement for Stenotic Bicuspid versus Tricuspid Aortic Valves: A Meta-Analysis and Meta-Regression

**DOI:** 10.1155/2019/8947204

**Published:** 2019-01-02

**Authors:** Raymundo A. Quintana, Dominique J. Monlezun, Adrian DaSilva-DeAbreu, Uday G. Sandhu, Derick Okwan-Duodu, Jonanlis Ramírez, Ali E. Denktas, Hani Jneid, David Paniagua

**Affiliations:** ^1^Division of Cardiology, Department of Medicine, Emory University School of Medicine, Atlanta, GA, USA; ^2^Division of Cardiology, Department of Medicine, The University of Texas MD Anderson Cancer Center, Houston, TX, USA; ^3^Division of Cardiology, John Ochsner Heart and Vascular Institute, New Orleans, LA, USA; ^4^Division of Cardiology, Department of Medicine, University of California San Francisco - Fresno, Fresno, CA, USA; ^5^Department of Pediatrics, Affiliated with Weil Cornell College of Medicine, Lincoln Medical and Mental Health Center, Bronx, NY, USA; ^6^Division of Cardiology, Department of Medicine, Baylor College of Medicine and Michael E. DeBakey VA Medical Center, Houston, TX, USA

## Abstract

**Objective:**

To assess 1-year mortality after transcatheter aortic valve replacement (TAVR) in patients with bicuspid aortic stenosis (AS).

**Background:**

Clinical trials have proven the beneficial effect of TAVR on mortality in patients with tricuspid AS. Individuals with bicuspid AS were excluded from these trials.

**Methods:**

A meta-analysis using literature search from the Cochrane, PubMed, ClinicalTrials, SCOPUS, and EMBASE databases was conducted to determine the effect of TAVR on 1-year mortality in patients with bicuspid AS. Short-term outcomes that could potentially impact one-year mortality were analyzed.

**Results:**

After evaluating 380 potential articles, 5 observational studies were selected. A total of 3890 patients treated with TAVR were included: 721 had bicuspid and 3,169 had tricuspid AS. No statistically significant difference between the baseline characteristics of the two groups of patients was seen outside of mean aortic gradient. Our primary endpoint of one-year all-cause mortality revealed 85 deaths in 719 patients (11.82%) with bicuspid AS compared to 467 deaths in 3100 patients (15.06%) with tricuspid AS, with no difference between both groups [relative risk (RR) 1.03; 95% CI 0.70-1.51]. Patients with bicuspid AS were associated with a decrease in device success (RR 0.62; 95% CI 0.45-0.84) and an increase in moderate-to-severe prosthetic valve regurgitation (RR 1.55; 95% CI 1.07-2.22) after TAVR compared to patients with tricuspid AS. The effect of meta-regression coefficients on one-year all-cause mortality was not statistically significant for any patient baseline characteristics.

**Conclusion:**

When comparing TAVR procedure in tricuspid AS versus bicuspid AS, there was no difference noted in one-year all-cause mortality.

## 1. Introduction

Transcatheter aortic valve replacement (TAVR) was first introduced in 2002 [1]. Since then, randomized controlled clinical trials have proven its beneficial effects on mortality, symptoms, and valve hemodynamics in patients with symptomatic severe aortic stenosis (AS) with native tricuspid aortic valve (TAV) [2–6]. Patients with bicuspid aortic valve (BAV), one of the most common congenital heart diseases, possess more severely calcified leaflets and raphe, asymmetric cusps, and dilated ascending aorta [7–9]. These features place patients with stenotic BAVs at a potentially higher risk of prosthetic valvular regurgitation and aortic dissection [8, 9]. Thus, patients with BAVs were excluded from all the key clinical trials and BAV has been considered a relative contraindication to TAVR [2–6]. The majority of the clinical outcomes data of TAVR in bicuspid AS come from small observational series [10–15].

The initial cohort of tricuspid AS TAVR patients was older and had more comorbidities than those with bicuspid AS, adding complexity to the outcomes comparison between both groups. However, with the expanding use of TAVR in intermediate surgical-risk patients, a direct comparison to bicuspid AS patients becomes feasible [4, 6, 16]. Despite the increasing off-label use of TAVR in stenotic BAVs, one-year outcomes in this patient population are currently unknown. Therefore, we conducted a meta-analysis and meta-regression of observational studies to evaluate mortality at one-year follow-up of TAVR in bicuspid AS patients and compare them with those of tricuspid AS patients. 

## 2. Methods

The current meta-analysis was conducted following the Preferred Reporting Items for Systematic Reviews and Meta-Analyses (PRISMA) guidelines [17].

### 2.1. Data Sources and Selection Criteria

**Figure 1:**
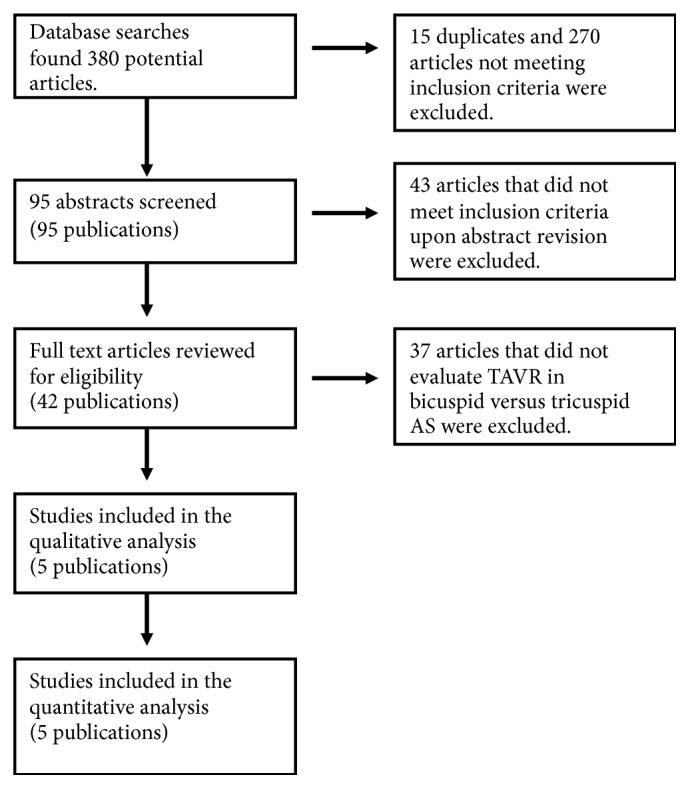
Results of search strategy.

A literature search was performed through the Cochrane, PubMed, ClinicalTrials, SCOPUS, and EMBASE databases from inception until January 2018. Original research manuscripts as well as conference abstracts with detailed information were included. The following search terms were used: “Transcatheter Aortic Valve Replacement” and “Bicuspid Aortic Valve.” No language restrictions were put into effect. References of retrieved articles and prior meta-analyses were searched for additional original research manuscripts and abstracts not encountered with our original search strategy.  Figure 1 illustrates the flowchart used for our research protocol according to PRISMA.

Studies included fulfilled the following criteria: (1) human studies of adults (age ≥18 years), (2) compared outcomes of patients undergoing TAVR who had bicuspid versus tricuspid AS, and (3) reported mortality at 1 year. Studies were excluded from the meta-analysis if the above criteria were not met or if the following specifications applied: (1) duplicate studies, (2) patient overlap among 2 studies or between abstracts and original research manuscripts, and (3) outcomes of interest being not reported. In case of potential patient overlap or duplicate studies, the most recent article with the largest patients number was included in our meta-analysis.

### 2.2. Data extraction and Quality Assessment

References and abstracts were screened and reviewed independently by two investigators (R.Q. and A.D.D.) to determine if inclusion criteria were met. Discrepancies were resolved by a third investigator (U.G.S.). These two investigators independently assessed the quality of the selected studies with the Critical Appraisal Skills Programme (CASP) tool for observational studies [18]. Once the articles to be included in the analysis were identified, data was extracted by ADD independently or in duplicate using a data collection tool developed specifically for this study. The Newcastle-Ottawa Scale was used to assess study quality.

### 2.3. Study Outcomes

Our primary endpoint was 1-year all-cause mortality. Secondary endpoints such as 30-day permanent pacemaker (PPM) implantation, moderate-to-severe prosthetic valve regurgitation, acute kidney injury and composite clinical endpoints 30-day safety, and device success defined in the Valve Academic Research Consortium 2 (VARC-2) were evaluated to detect early differences between both groups which could impact one-year mortality [19]. Short-term outcomes have been previously pooled and reported in meta-analysis by others; thus our literature review was designated to evaluate one-year mortality [20].

### 2.4. Data Analysis

For dichotomous data, risk ratios with 95% confidence intervals (CIs) were used to summarize the study results. For continuous variables, standardized mean differences (SMD) with 95% CIs were used. A random-effects model was chosen over fixed-effects model to estimate the average treatment effect based on the assumption of differences in the treatment effect and/or sampling variability between studies; this assumption would be tested with Cochran's Q-test (p value < 0.1) and I^2^ statistic for heterogeneity expressed as a percentage. Publication bias was evaluated using a funnel plot and further quantified with Begg's test for small-study effects, considering statistically significant corrected p value of less than 0.05. The effects of missing data in the main outcome were explored using sensitivity analysis with best-case analysis and worst-case analysis. Meta-regression analyses investigating the effects of study-level characteristics including diabetes mellitus, hypertension, heart failure with New York Heart Association (NYHA) symptoms class III-IV, CKD (GFR <60 ml/min/1.73 m^2^), previous cerebrovascular accidents, female gender, CAD, PAD, COPD, prior PCI, and prior CABG on 1-year mortality after TAVR were conducted using the variables as proportions. Mean age, mean LVEF, STS score, EuroScore, and mean aortic gradient are represented in their respective standard continuous units. We used the baseline patient traits from the individual studies as independent variables in linear meta-regression on the log-transformed RR of BAV versus TAV on one-year mortality to calculate the variables' meta-regression coefficients with 95% CIs, thus testing if any of the variables were modulators of the effect of BAV versus TAV on mortality. Statistical analysis was performed using Stata version 14.2 (StataCorp LP, College Station, Texas).

## 3. Results

Literature search revealed 380 potential articles and abstracts. After the removal of duplicates and screening of references, abstracts, and full texts, 5 observational studies were selected and included in our analysis (Figure 1). These studies fulfilled all the established inclusion criteria and were fully written in English language [10, 11, 21–23]. Baseline characteristics of the studied patient populations with relative risks/SMDs are described in Table 1. A total of 3890 patients treated with TAVR were included in these studies: 721 had bicuspid and 3169 had tricuspid aortic stenosis. Except for a greater mean aortic valve gradient in the BAV group compared to the TAV group [SMD 0.10 (0.00-0.20; p=0.048)], there was no statistically significant difference in the baseline characteristics between both groups (Table 1).

### 3.1. Primary Endpoint

**Figure 2:**
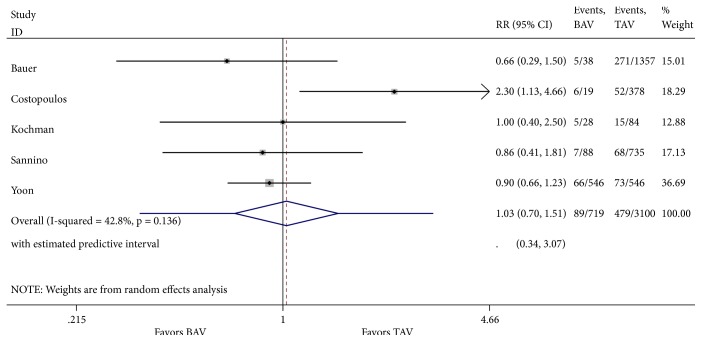
Forest plot of random-effects model of 1-year mortality following TAVR in patients with bicuspid versus tricuspid aortic valve. Heterogeneity for this outcome was nonsignificant (I^2^ = 42.8%; p=0.136).

**Figure 3:**
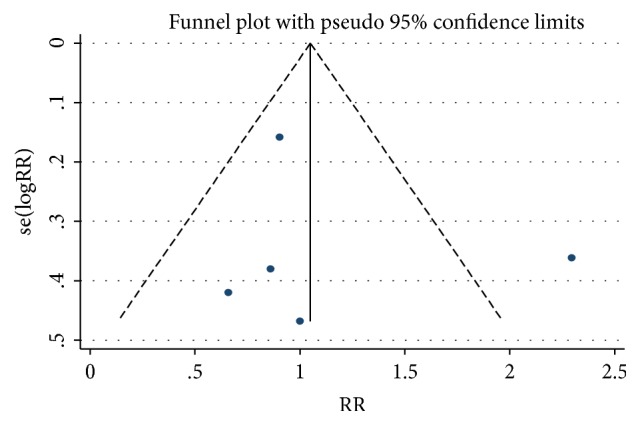
Funnel plot of included trials. Begg's test for small-study effects was not statistically significant (p=0.09).

Analysis of one-year all-cause mortality revealed 85 deaths in 719 patients (11.82%) with severe bicuspid AS compared to 467 deaths in 3100 patients (15.06%) with tricuspid AS. There was no difference in 1-year all-cause mortality between both groups [relative risk (RR) 1.03; 95% CI 0.70-1.51, with no significant overall heterogeneity between studies (Figure 2). There was no evidence of publication bias in funnel plot analysis and Begg's test for small-study effects was consistent with this finding (p = 0.09) (Figure 3). There were missing data for one of the included studies [11], with 2 patients lost to follow-up in the bicuspid group and 69 in the tricuspid group. Sensitivity analysis was performed with best-case and worst-case analysis. In neither cases, the RR was significantly different than 1 (0.96; 95% CI 0.75-1.24 and 1.17; 95% CI 0.68-2.02, resp.). Heterogeneity was significant in worst-case analysis. Subgroup meta-analysis performed by missing cases revealed minimal heterogeneity among studies with no missing data.

### 3.2. Secondary Endpoints

**Figure 4:**
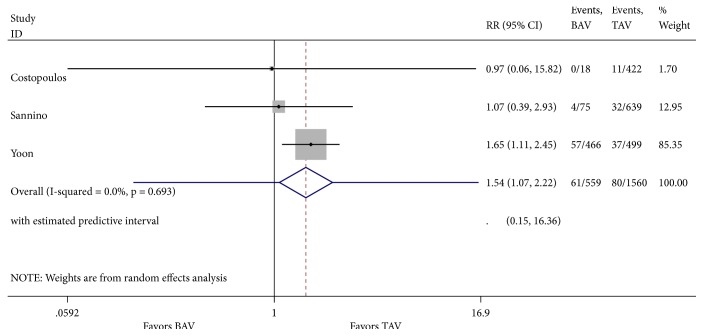
Forest plot for 30-day post-TAVR moderate-to-severe prosthetic valve regurgitation.

**Figure 5:**
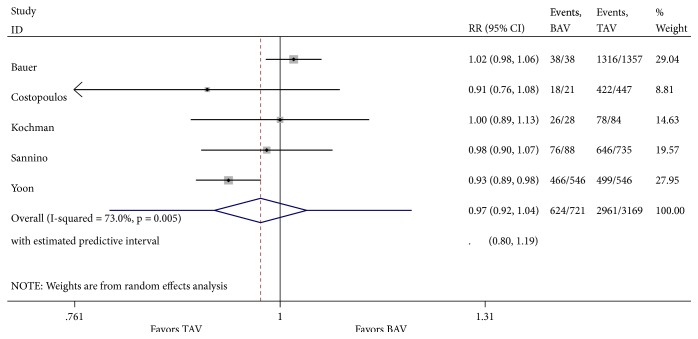
Forest plot for 30-day post-TAVR composite device success.

**Figure 6:**
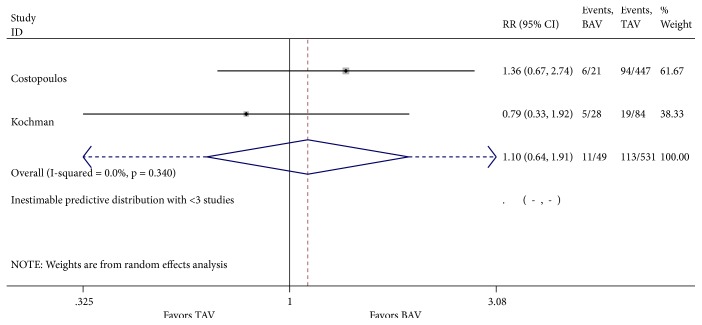
Forest plot for 30-day post-TAVR composite safety.

**Figure 7:**
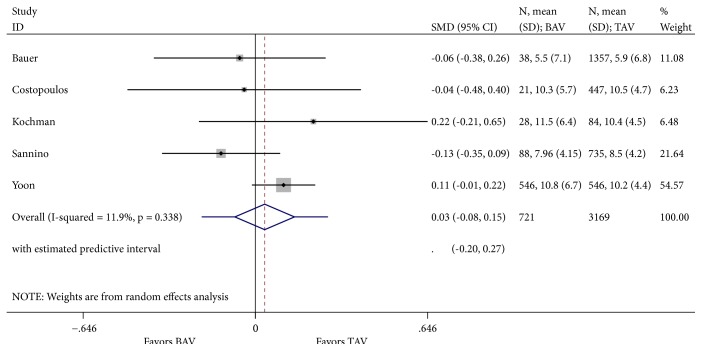
Forest plot for 30-day post-TAVR mean aortic gradient.

**Figure 8:**
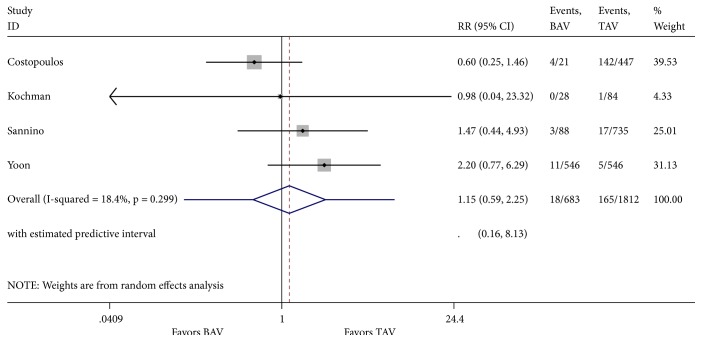
Forest plot for 30-day post-TAVR acute kidney injury.

**Figure 9:**
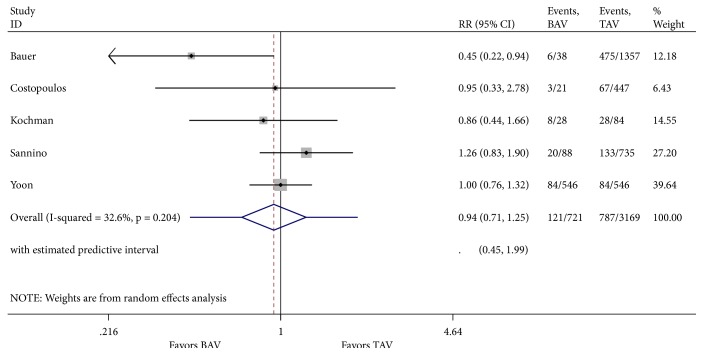
Forest plot for 30-day post-TAVR permanent pacemaker implantation.

Our literature review was designated to evaluate one-year mortality. However, analysis of cardinal short-term outcomes that could potentially have an impact on all-cause one-year mortality in both groups of patients was conducted and summarized in Table 2. We found that TAVR in patients with BAV was associated with a significant increase in new moderate-to-severe prosthetic valve regurgitation (RR 1.55; 95% CI 1.07-2.22, I^2^ = 0%) when compared to patients with TAV (Figure 4). Device success was appreciated in 621 of 721 (86.5%) and in 2961 of 3169 (93.4%) TAVR cases in the BAV and TAV group, respectively. TAVR in patients with BAV was associated with a significant decrease in device success (RR 0.62; 95% CI 0.45-0.85, I^2^ = 0%) (Figure 5). At 30-day follow-up, there was no significant difference in the composite safety endpoint which occurred in 22.45% of patients with BAV compared to 21.28% of patients with TAV undergoing TAVR (RR 1.10; 95% CI 0.64-1.91 and I^2^ = 0%) (Figure 6). There was no significant difference in the post-TAVR mean aortic gradient (SMD 0.03; 95% CI -0.08-0.15, I^2^ =11.9%) (Figure 7), acute kidney injury (RR 0.24; 95% CI 0.02-2.58, I^2^ =92.3%) (Figure 8), and permanent pacemaker implantation (RR 0.94; 95% CI 0.71-1.25, I^2^ =32.6%) (Figure 9) between both groups. Funnel plots for 30-day outcomes are depicted in supplementary Figures S1-S6.

### 3.3. Meta-Regression

The effects of meta-regression coefficients on one-year all-cause mortality were not statistically significant for mean age, diabetes mellitus, hypertension, heart failure with New York Heart Association (NYHA) symptoms class III-IV, CKD (GFR <60 ml/min/1.73 m^2^), mean LVEF, previous cerebrovascular accidents, female gender, CAD, PAD, COPD, STS score, EuroScore, mean aortic gradient, prior PCI, and prior CABG (Table 3). Meta-regression representative bubble plots are shown in Figures S7-S22.

## 4. Discussion

To our knowledge, these are the first meta-analysis and meta-regression analysis of 1-year outcomes in patients with severe bicuspid AS undergoing TAVR. We found no significant difference in 1-year mortality between patients with BAV (11.82%) and TAV (15.06%) undergoing TAVR (RR 1.03; 95% CI 0.70-1.51), which persisted when performing a best-case and worst-case analysis of missing data. This supports the fact that the lost to follow-up patients in one of the included studies did not affect the outcomes of our analysis [11]. We did find moderate between-studies heterogeneity in our analysis; however, the minimal heterogeneity among studies with no missing data suggests that the main source of heterogeneity is due to missing data from the study by Costopoulos et al. [11]. Also, there was no evidence of publication bias to suggest our results to be affected by lack of published studies.

Previous studies found stroke, acute kidney, myocardial infarction, and a LVEF <30% as strong predictors of mortality after TAVR in patients with TAVs [24, 25]. Identifying the clinical characteristics that impact patient mortality after TAVR is of cardinal importance for the development of appropriate preventive strategies in patients with BAVs. Thus, we conducted a meta-regression analysis to assess if the effects of BAV versus TAV on 1-year all-cause mortality were affected by patient's baseline characteristics. We found that none of these variables modulate 1-year all-cause mortality. Further larger studies should be conducted to confirm our findings.

When comparing these findings with data from the STS/ACC TVT Registry for TAVR in TAVs, which documented an all-cause 1-year mortality of 21.6%, we found a lower rate of mortality at 1 year in both groups (BAV and TAV group) of this study [26]. We believe these results could be explained by the smaller sample size, healthier participants of the included studies, and the greater proportion of first-generation devices in the STS/ACC TVT Registry. New-generation prosthetic valves allow for improved positioning, effectively decreasing major vascular complications and prosthetic valve regurgitation, which can have a positive impact on one-year mortality [26]. The impact of different TAVR approach, bicuspid valve morphology, or prosthetic valve type on outcomes was not assessed in our study.

With regard to short-term outcomes, we found a significantly higher rate of moderate-to-severe prosthetic valve regurgitation and decreased device success in patients with BAV. Recent data has shown that BAVs do not possess a more elliptical annulus compared to TAVs [27]. However, other anatomical features more prevalent in bicuspid AS such as severely calcified leaflets, asymmetric cusps, and concomitant aortopathy have been associated with incomplete prosthesis apposition, misdeployment, prosthetic valve regurgitation, and device failure [9, 28, 29]. Thus, an accurate aortic annulus sizing for prosthesis selection is imperative in this patient population and frequently requires the use of multimodality imaging including transesophageal echocardiography, 3D computed tomography, and calibrated balloon valvuloplasty [30].

In our study, a higher rate of moderate-to-severe prosthetic valve regurgitation and decreased device success had no impact on one-year all-cause mortality. A recent meta-analysis reported similar findings and also a significantly increased conversion to open aortic valve in patients with BAV after TAVR [31]. Thus, a possible explanation for the lack of difference in one-year mortality between both groups could be the early diagnosis and surgical treatment of moderate-to-severe prosthetic valve regurgitation before cardiac remodeling and clinical deterioration occur. Although an increased rate of permanent pacemaker implantation has been described in patients with BAVs compared to those with TAVs [27], we did not find a difference in permanent pacemaker implantation between both groups.

The small number of patients in each study and the observational nature of studies evaluating one-year outcomes in patients with bicuspid AS undergoing TAVR are limitations of our analysis. The included studies were conducted in multiple countries/continents, with different valvular devices and equipment. Thus, outcome reporting by the investigators was variable. Although the observational nature of the studies included carries an inherent risk of bias, all of the included studies had a Newcastle-Ottawa scale of 7 or greater (of a maximun score of 8), which supports the fact that the included observational studies were of adequate quality.

## 5. Conclusion

In conclusion, our findings support the use of TAVR in appropriately selected patients with severe symptomatic bicuspid AS. Despite a decreased device success and moderate-to-severe prosthetic valve regurgitation, this technique was found to be safe without an associated 1-year increased mortality in patients with BAV.

## Figures and Tables

**Table 1:** Baseline characteristics and pooled rates data.

**First author**	**Valve type**	**Bauer 2014**	**Costopoulos 2014**	**Kochman 2014**	**Sannino 2017**	**Yoon 2017**	**Relative risk (95**%**CI; p value)**
**# of patients**	BAV	38	21	28	88	546	NA
	TAV	1357	447	84	735	546	

**Devices**	BAV	CV, Sapien	CV, Sapien	CV, Sapien	SXT, S3, CV, ER, Lotus	SXT, S3, CV, ER, Lotus	NA
	TAV	CV, Sapien	CV, Sapien	CV, Sapien	SXT, S3, CV, ER, Lotus	SXT, S3, CV, ER, Lotus	

**Approaches**	BAV	TA, TAo, TF	TF, TA, TAx	TF, TA, TAo, TS	TF, TA, TAo, TS	TF	NA
	TAV	TA, TAo, TF, TAx	TF, TA, TAo, TAx	TF, TA, TAo, TS	TF, TA, TAo, TS	TF	

**STS Score, **%	BAV	-	7.6 ± 4.2	-	7.4 ± 3.9	4.6 ± 4.6	0.04 (-0.06, 0.15; p=0.393)^*∗*^
	TAV	-	7.8 ± 7.3	-	7.6 ± 3.9	4.3 ± 3.0	

**EuroScore, **%	BAV	18 ± 10	23.9 ± 12.0	19.2 ± 9.0	-	16.1 ± 12.0	-0.07 (-0.17, 0.04; p=0.222)^*∗*^
	TAV	20 ± 13	24.4 ± 17.3	18.8 ± 8.7	-	16.9 ± 13.9	

**Mean age in years**	BAV	80.7 ± 6.6	76.7 ± 7.1	77.6 ± 5.5	80.2 ± 8.4	77.2 ± 8.2	-0.09 (-0.21, 0.03; p=0.156)^*∗*^
	TAV	81.8 ± 6.2	79.8 ± 7.4	79.1 ± 6.8	81.8 ± 7.9	77.2 ± 8.8	

**Females**	BAV	55.3	42.9	53.6	39.8	37.2	0.92 (0.82, 1.03; p=0.144)
	TAV	58.0	52.6	57.1	47.1	39.4	

**Mean aortic gradient**	BAV	-	54.4 ± 17.9	55.5 ± 17.6	46.9 ± 16.9	49.7 ± 17.7	0.10 (0.00, 0.20; p=0.048)^*∗*^
	TAV	-	52.5 ± 16.0	52.5 ± 18.9	44.3 ± 13.6	48.5 ± 17.1	

**Mean LVEF**	BAV	50 ± 16	50.1 ± 12.4	48.1 ± 13.1	-	51.6 ± 15.0	-0.04 (-0.14, 0.07; p=0.489)^*∗*^
	TAV	53 ± 15	52.0 ± 12.6	49.8 ± 14.0	-	51.6 ± 15.2	

**NYHA III/IV**	BAV	84.2	71.4	71.4	-	-	0.96 (0.86, 1.07; p=0.438)
	TAV	89.0	67.3	78.6	-	-	

**HTN**	BAV	-	66.7	60.7	80.7	70.0	0.98 (0.92, 1.04; p=0.457)
	TAV	-	77.2	65.5	83.3	70.5	

**DM**	BAV	36.8	28.6	39.3	33.0	23.4	1.20 (0.80, 1.79, p=0.372)
	TAV	34.0	30.2	34.5	38.5	23.3	

**COPD**	BAV	21.1	33.3	21.4	17.0	-	1.00 (0.74, 1.36; p=0.979)
	TAV	24.0	30.6	20.2	20.3	-	

**CAD**	BAV	68.4	-	50.0	69.3	-	1.04 (0.89, 1.21; p=0.631)
	TAV	60.0	-	64.3	66.3	-	

**PAD**	BAV	10.5	33.3	21.4	39.8	15.2	0.99 (0.73, 1.33; p=0.929)
	TAV	22.0	29.8	34.5	29.8	15.6	

**Prior PCI**	BAV	34.2	28.6	21.4	-	22.2	0.95 (0.79, 1.14; p=0.569)
	TAV	35.0	21.5	35.7	-	23.4	

**CKD (GFR <60 ml/min)**	BAV	22	11	12	45	-	1.03 (0.89, 1.20; p=0.665)
	TAV	828	257	36	330	-	

**Prior CABG**	BAV	13.2	14.3	14.3	-	11.4	0.85 (0.65, 1.13; p=0.258)
	TAV	18.0	19.9	25.0	-	12.3	

**Previous cerebrovascular accident**	BAV	13.2	19.0	28.6	19.3	14.1	1.18 (0.95, 1.48; p=0.137)
	TAV	8.0	16.1	16.7	18.0	12.6	

*∗* = Standardized Mean Difference (SMD).

P value for test of SDM = 0 or relative risk = 1 as appropriate.

BAV = Bicuspid aortic valve; CABG = Coronary artery bypass graft; CAD = Coronary artery disease; CKD = Chronic kidney disease; COPD = Chronic obstructive pulmonary disease; CV = Core Valve; DM = Diabetes mellitus; ER = CoreValve Evolut R; EuroScore = European System for Cardiac Operative Risk Evaluation; GFR = Glomerular filtration rate; HTN = Hypertension; LVEF = Left ventricular ejection fraction; NYHA = New York Heart Association; PAD = Peripheral artery disease; PCI = Percutaneous coronary intervention; S3 = Sapien S3; STS = Society of Thoracic Surgeons; S = Sapien; SXT = Sapien XT; TA = Transapical; TAo = Transaortic; TAV = Tricuspid aortic valve; TAx = Transaxillary; TF = Transfemoral; TS = Transubclavian.

**Table 2:** Short-term outcomes after TAVR for patients with bicuspid versus tricuspid (Reference) aortic valves.

**Short-term outcome**	**Proportion of patients in the BAV group**	**Proportion of patients in the TAV group**	**Relative Risk or Standardized Mean Difference**	**Estimate**	**95**%** Confidence Interval**	**Heterogenicity** **I**^**2**^**(**%**)**
Moderate-severe prosthetic valve regurgitation	61/559	80/1560	RR	1.54	1.07-2.22	0

Device success	624/721	2961/3169	RR	0.62	0.45-0.84	0

Composite Safety	11/49	113/531	RR	1.1	0.64-1.91	0

Mean Aortic Gradient	721	3169	SMD	0.03	-0.08-0.15	11.9

Acute Kidney Injury	18/683	165/1812	RR	0.24	0.059-2.25	18.4

Permanent Pacemaker Implantation	121/721	787/3169	RR	0.94	0.71-1.25	32.6

BAV = bicuspid aortic valve; TAV = tricuspid aortic valve; RR = Relative Risk; SMD = Standardized Mean Difference.

**Table 3:** Meta regression analyses.

**Baseline characteristic**	**Mean regression coefficient**	**95**%** CI**	**P-Value**
Mean Age	-0.04	-0.38 - 0.29	0.652
Diabetes Mellitus	-1.12	-15.52 - 13.28	0.821
Hypertension	1.06	-18.30 - 20.42	0.836
NYHA III-IV	-6.25	-40.09 - 27.58	0.256
Chronic Kidney Disease (GFR <60 ml/min/1.73 m^2^)	0.95	-19.91 - 21.82	0.862
Mean LVEF	-0.10	-1.32 - 1.11	0.748
Previous cerebrovascular accident	4.42	-14.73 - 23.57	0.516
Female Gender	-0.41	-11.75 - 10.93	0.916
CAD	-1.67	-73.48 - 70.15	0.817
PAD	2.62	-5.66 - 10.90	0.388
COPD	8.75	-6.23 - 23.72	0.129
STS Score	0.17	-3.11 - 3.44	0.637
EuroScore	0.12	-0.14 - 0.39	0.180
Mean Aortic Gradient	0.09	-0.21, 0.40	0.324
Prior PCI	-5.00	-35.41 - 25.40	0.552
Prior CABG	5.38	-41.60 - 52.37	0.671

CABG = Coronary artery bypass graft; CAD = Coronary artery disease; CKD = Chronic kidney disease; COPD = Chronic obstructive pulmonary disease; EuroScore = European System for Cardiac Operative Risk Evaluation; GFR = Glomerular filtration rate; LVEF = Left ventricular ejection fraction; NYHA = New York Heart Association; PAD = Peripheral artery disease; PCI = Percutaneous coronary intervention; STS = Society of Thoracic Surgeons.

## Data Availability

All data generated or analyzed in order to support the findings of this study are included within the article and the supplementary information file.

## Supplementary Materials

Supplementary Materials Figure S1: assessment of publication bias by funnel plot of included articles for 30-day post-TAVR moderate-to-severe prosthetic valve regurgitation in bicuspid versus tricuspid aortic stenosis. There is no conclusive evidence of publication bias. Figure S2: assessment of publication bias by funnel plot of included articles for 30-day post-TAVR composite device success in bicuspid versus tricuspid aortic stenosis. There is no conclusive evidence of publication bias. Figure S3: assessment of publication bias by funnel plot of included articles for 30-day post-TAVR composite safety in bicuspid versus tricuspid aortic stenosis. There is no conclusive evidence of publication bias. Figure S4: assessment of publication bias by funnel plot of included articles for 30-day post-TAVR mean aortic gradient in bicuspid versus tricuspid aortic stenosis. There is no conclusive evidence of publication bias. Figure S5: assessment of publication bias by funnel plot of included articles for 30-day post-TAVR acute kidney injury in bicuspid versus tricuspid aortic stenosis. There is no conclusive evidence of publication bias. Figure S6: assessment of publication bias by funnel plot of included articles for 30-day post-TAVR permanent pacemaker implantation in bicuspid versus tricuspid aortic stenosis. There is no conclusive evidence of publication bias. Figure S7: meta-regression bubble plot of the relationship of mean age with the logarithm of a relative risk for one-year all-cause mortality after TAVR. The regression line (red line) is shown. The size of the circle represents the weighting of each observational study and is inversely proportional to the standard error of the effect estimate. The result of meta-regression analysis failed to demonstrate a significant relationship between mean age and one-year all-cause mortality after TAVR (p = 0.652). Figure S8: meta-regression bubble plot of the relationship of diabetes mellitus with the logarithm of a relative risk for one-year all-cause mortality after TAVR. The regression line (red line) is shown. The size of the circle represents the weighting of each observational study and is inversely proportional to the standard error of the effect estimate. The result of meta-regression analysis failed to demonstrate a significant relationship between diabetes mellitus and one-year all-cause mortality after TAVR (p = 0.821). Figure S9: meta-regression bubble plot of the relationship of hypertension with the logarithm of a relative risk for one-year all-cause mortality after TAVR. The regression line (red line) is shown. The size of the circle represents the weighting of each observational study and is inversely proportional to the standard error of the effect estimate. The result of meta-regression analysis failed to demonstrate a significant relationship between hypertension and one-year all-cause mortality after TAVR (p = 0.836). Figure S10: meta-regression bubble plot of the relationship of heart failure with NYHA III-IV symptoms with the logarithm of a relative risk for one-year all-cause mortality after TAVR. The regression line (red line) is shown. The size of the circle represents the weighting of each observational study and is inversely proportional to the standard error of the effect estimate. The result of meta-regression analysis failed to demonstrate a significant relationship between heart failure with NYHA III-IV symptoms and one-year all-cause mortality after TAVR (p = 0.256). Figure S11: meta-regression bubble plot of the relationship of chronic kidney disease (GFR <60 ml/min/1.73 m^2^) with the logarithm of a relative risk for one-year all-cause mortality after TAVR. The regression line (red line) is shown. The size of the circle represents the weighting of each observational study and is inversely proportional to the standard error of the effect estimate. The result of meta-regression analysis failed to demonstrate a significant relationship between chronic kidney disease and one-year all-cause mortality after TAVR (p = 0.862). Figure S12: meta-regression bubble plot of the relationship of mean left ventricle ejection fraction (LVEF, %) with the logarithm of a relative risk for one-year all-cause mortality after TAVR. The regression line (red line) is shown. The size of the circle represents the weighting of each observational study and is inversely proportional to the standard error of the effect estimate. The result of meta-regression analysis failed to demonstrate a significant relationship between LVEF and one-year all-cause mortality after TAVR (p = 0.748). Figure S13: meta-regression bubble plot of the relationship of previous cerebrovascular accident (CVA) with the logarithm of a relative risk for one-year all-cause mortality after TAVR. The regression line (red line) is shown. The size of the circle represents the weighting of each observational study and is inversely proportional to the standard error of the effect estimate. The result of meta-regression analysis failed to demonstrate a significant relationship between CVA and one-year all-cause mortality after TAVR (p = 0.516). Figure S14: meta-regression bubble plot of the relationship of female gender with the logarithm of a relative risk for one-year all-cause mortality after TAVR. The regression line (red line) is shown. The size of the circle represents the weighting of each observational study and is inversely proportional to the standard error of the effect estimate. The result of meta-regression analysis failed to demonstrate a significant relationship between female gender and one-year all-cause mortality after TAVR (p = 0.916). Figure S15: meta-regression bubble plot of the relationship of STS score with the logarithm of a relative risk for one-year all-cause mortality after TAVR. The regression line (red line) is shown. The size of the circle represents the weighting of each observational study and is inversely proportional to the standard error of the effect estimate. The result of meta-regression analysis failed to demonstrate a significant relationship between STS score and one-year all-cause mortality after TAVR (p = 0.637). Figure S16: meta-regression bubble plot of the relationship of EuroScore with the logarithm of a relative risk for one-year all-cause mortality after TAVR. The regression line (red line) is shown. The size of the circle represents the weighting of each observational study and is inversely proportional to the standard error of the effect estimate. The result of meta-regression analysis failed to demonstrate a significant relationship between EuroScore and one-year all-cause mortality after TAVR (p = 0.180). Figure S17: meta-regression bubble plot of the relationship of pre-TAVR mean aortic gradient with the logarithm of a relative risk for one-year all-cause mortality after TAVR. The regression line (red line) is shown. The size of the circle represents the weighting of each observational study and is inversely proportional to the standard error of the effect estimate. The result of meta-regression analysis failed to demonstrate a significant relationship between pre-TAVR mean aortic gradient and one-year all-cause mortality after TAVR (p = 0.324). Figure S18: meta-regression bubble plot of the relationship of chronic obstructive pulmonary disease (COPD) with the logarithm of a relative risk for one-year all-cause mortality after TAVR. The regression line (red line) is shown. The size of the circle represents the weighting of each observational study and is inversely proportional to the standard error of the effect estimate. The result of meta-regression analysis failed to demonstrate a significant relationship between COPD and one-year all-cause mortality after TAVR (p = 0.129). Figure S19: meta-regression bubble plot of the relationship of coronary artery disease (CAD) with the logarithm of a relative risk for one-year all-cause mortality after TAVR. The regression line (red line) is shown. The size of the circle represents the weighting of each observational study and is inversely proportional to the standard error of the effect estimate. The result of meta-regression analysis failed to demonstrate a significant relationship between CAD and one-year all-cause mortality after TAVR (p = 0.817). Figure S20: meta-regression bubble plot of the relationship of peripheral artery disease (PAD) with the logarithm of a relative risk for one-year all-cause mortality after TAVR. The regression line (red line) is shown. The size of the circle represents the weighting of each observational study and is inversely proportional to the standard error of the effect estimate. The result of meta-regression analysis failed to demonstrate a significant relationship between PAD and one-year all-cause mortality after TAVR (p = 0.388). Figure S21: meta-regression bubble plot of the relationship of percutaneous coronary intervention (PCI) with the logarithm of a relative risk for one-year all-cause mortality after TAVR. The regression line (red line) is shown. The size of the circle represents the weighting of each observational study and is inversely proportional to the standard error of the effect estimate. The result of meta-regression analysis failed to demonstrate a significant relationship between PCI and one-year all-cause mortality after TAVR (p = 0.552). Figure S22: meta-regression bubble plot of the relationship of coronary artery bypass graft (CABG) with the logarithm of a relative risk for one-year all-cause mortality after TAVR. The regression line (red line) is shown. The size of the circle represents the weighting of each observational study and is inversely proportional to the standard error of the effect estimate. The result of meta-regression analysis failed to demonstrate a significant relationship between CABG and one-year all-cause mortality after TAVR (p = 0.671).
