# Supplementary material for: One-Year Mortality in Patients Undergoing Transcatheter Aortic Valve Replacement for Stenotic Bicuspid versus Tricuspid Aortic Valves: A Meta-Analysis and Meta-Regression
Source: J Interv Cardiol. 2019 Jan 2;2019:8947204. doi: 10.1155/2019/8947204 (PMC6739767; doi:10.1155/2019/8947204)
Supplement: Supplementary Materials — Figure S1: assessment of publication bias by funnel plot of included articles for 30-day post-TAVR moderate-to-severe prosthetic valve regurgitation in bicuspid versus tricuspid aortic stenosis. There is no conclusive evidence of publication bias. Figure S2: assessment of publication bias by funnel plot of included articles for 30-day post-TAVR composite device success in bicuspid versus tricuspid aortic stenosis. There is no conclusive evidence of publication bias. Figure S3: assessment of publication bias by funnel plot of included articles for 30-day post-TAVR composite safety in bicuspid versus tricuspid aortic stenosis. There is no conclusive evidence of publication bias. Figure S4: assessment of publication bias by funnel plot of included articles for 30-day post-TAVR mean aortic gradient in bicuspid versus tricuspid aortic stenosis. There is no conclusive evidence of publication bias. Figure S5: assessment of publication bias by funnel plot of included articles for 30-day post-TAVR acute kidney injury in bicuspid versus tricuspid aortic stenosis. There is no conclusive evidence of publication bias. Figure S6: assessment of publication bias by funnel plot of included articles for 30-day post-TAVR permanent pacemaker implantation in bicuspid versus tricuspid aortic stenosis. There is no conclusive evidence of publication bias. Figure S7: meta-regression bubble plot of the relationship of mean age with the logarithm of a relative risk for one-year all-cause mortality after TAVR. The regression line (red line) is shown. The size of the circle represents the weighting of each observational study and is inversely proportional to the standard error of the effect estimate. The result of meta-regression analysis failed to demonstrate a significant relationship between mean age and one-year all-cause mortality after TAVR (p = 0.652). Figure S8: meta-regression bubble plot of the relationship of diabetes mellitus with the logarithm of a relative risk for one-year all-ca [file 8947204.f1.pdf]

Supplemental Material:

Funnel plots for 30 day outcomes:

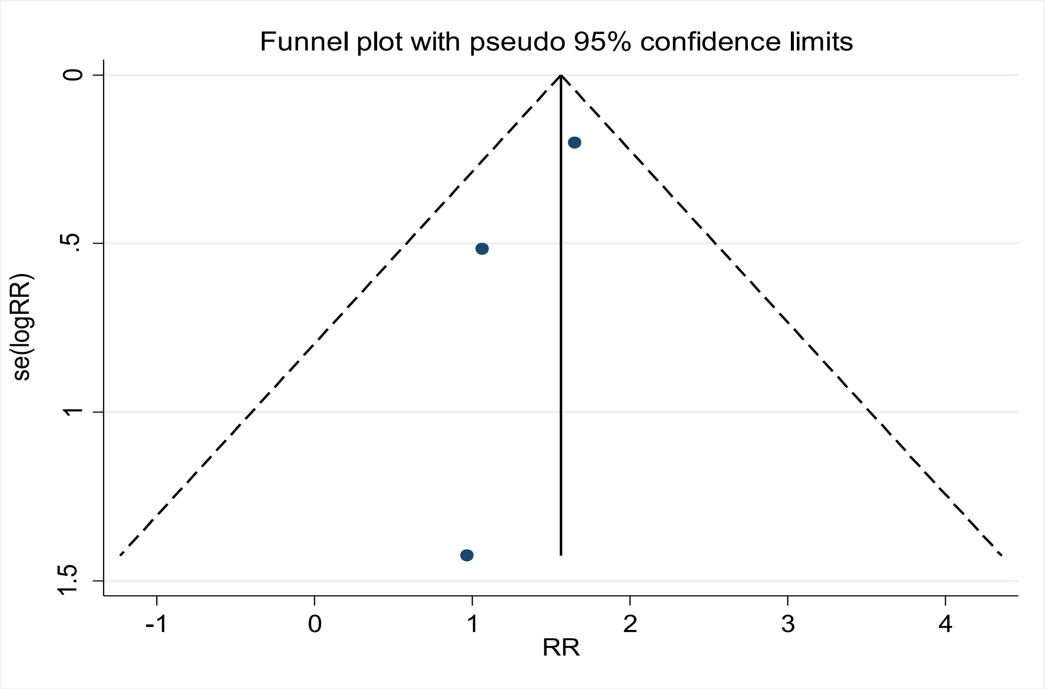

Figure S1 Funnel plot for 30-day Post-TAVR moderate to severe prosthetic valve regurgitation

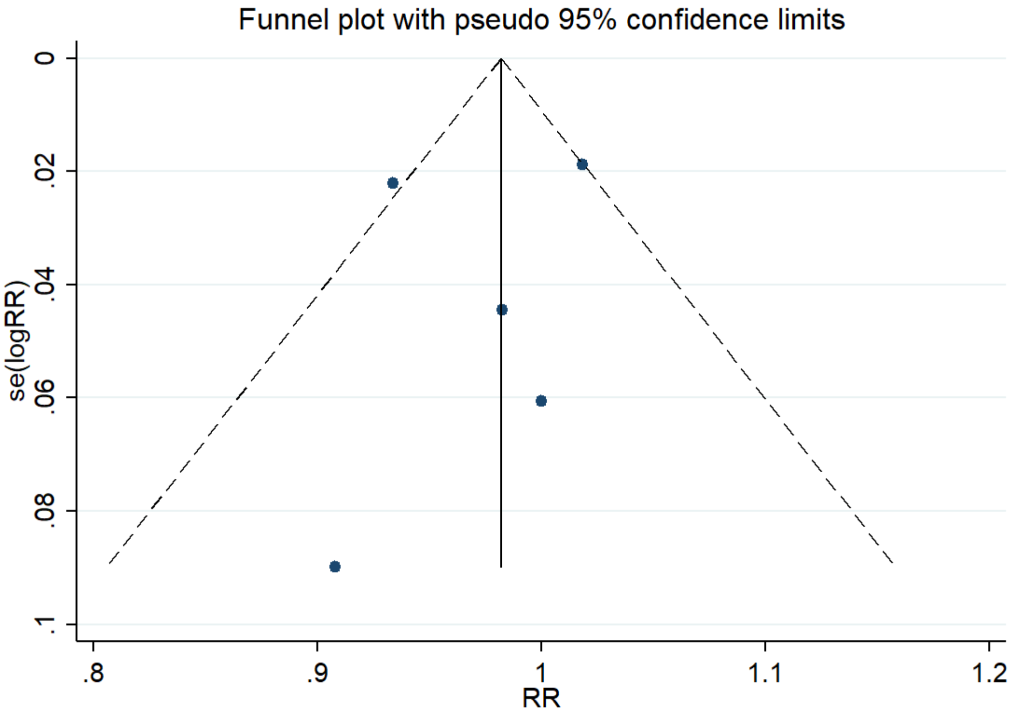

Figure S2 Funnel plot for 30-day Post-TAVR composite device success

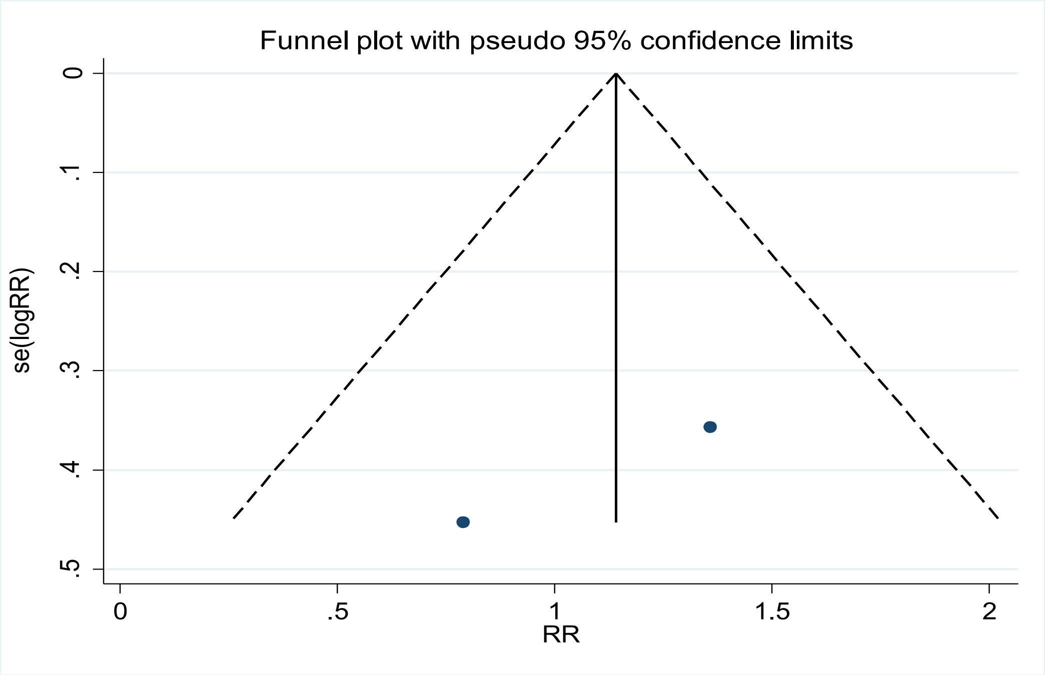

Figure S3 Funnel plot for 30-day Post-TAVR composite safety

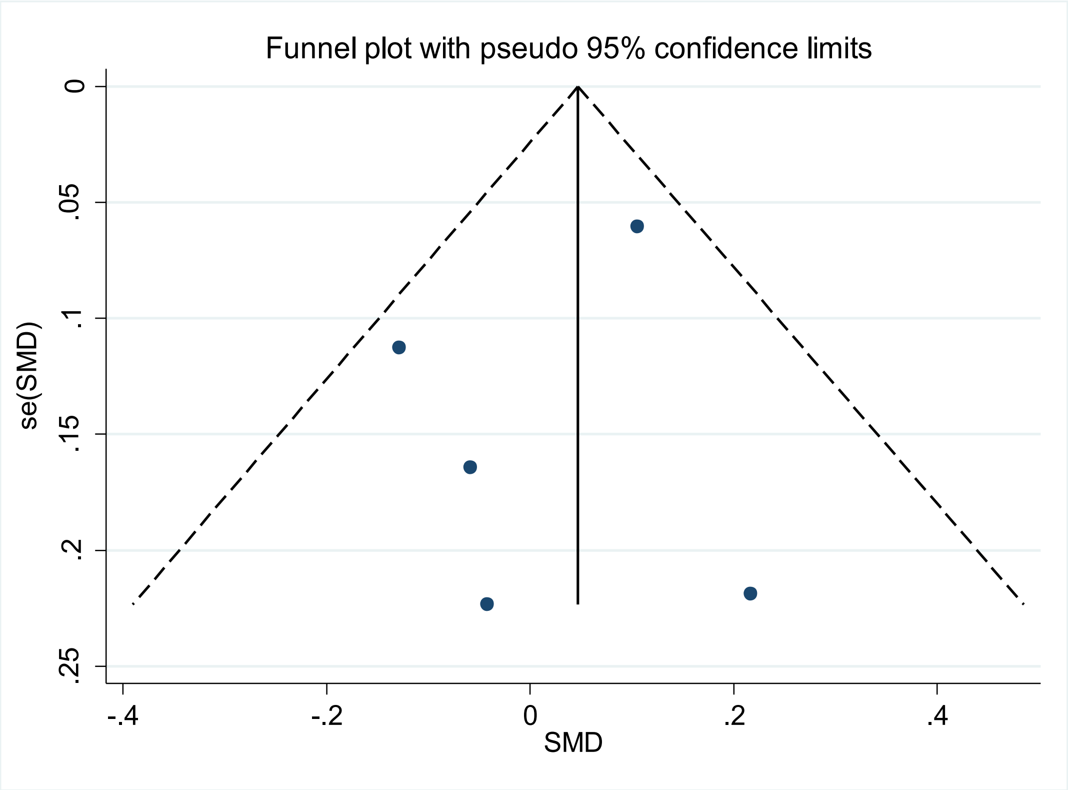

Figure S4 Funnel plot for 30-day Post-TAVR mean aortic gradient

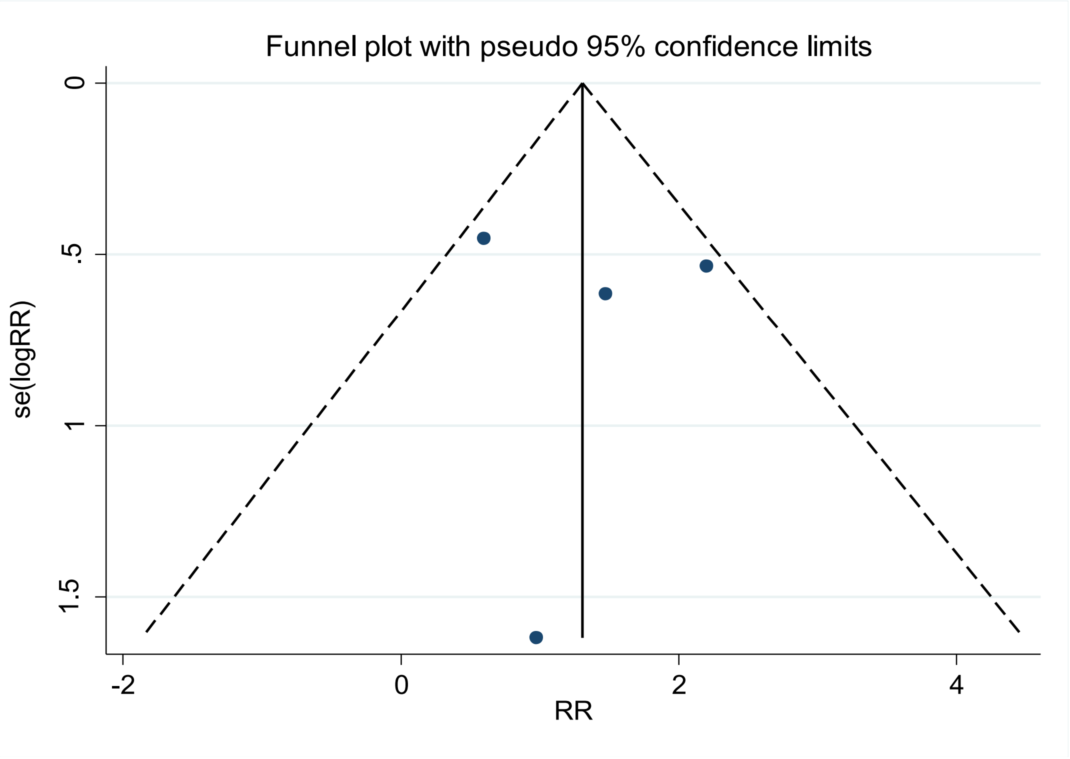

Figure S5 Funnel plot for 30-day Post-TAVR acute kidney injury

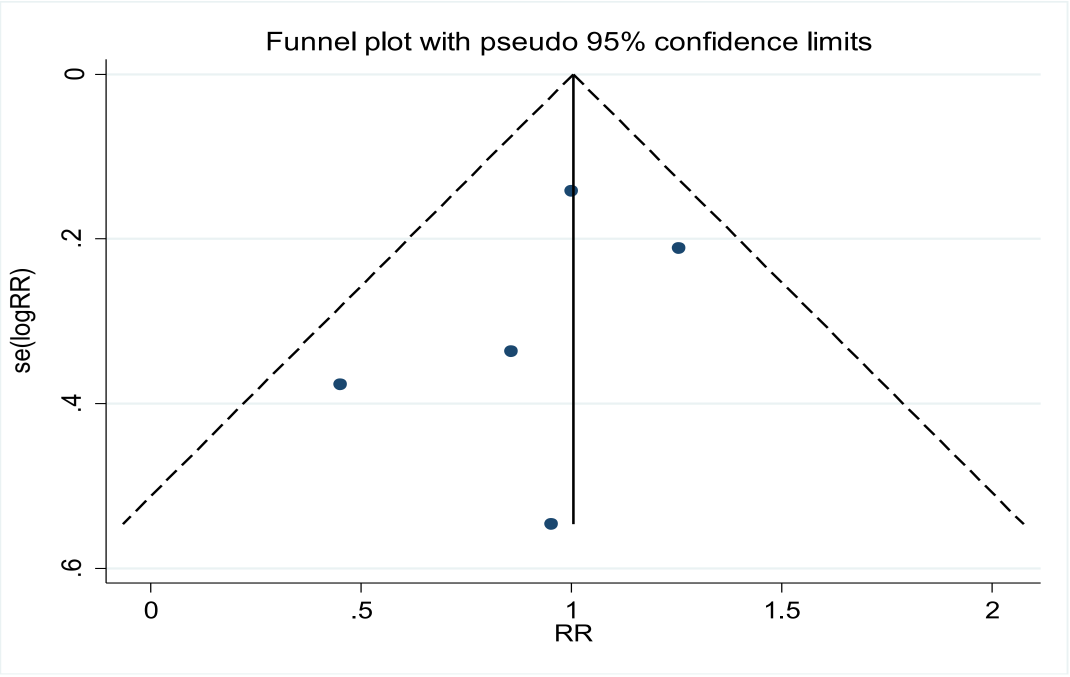

Figure S6 Funnel plot for 30-day Post-TAVR permanent pacemaker implantation

Meta-regression analysis by representative plots:

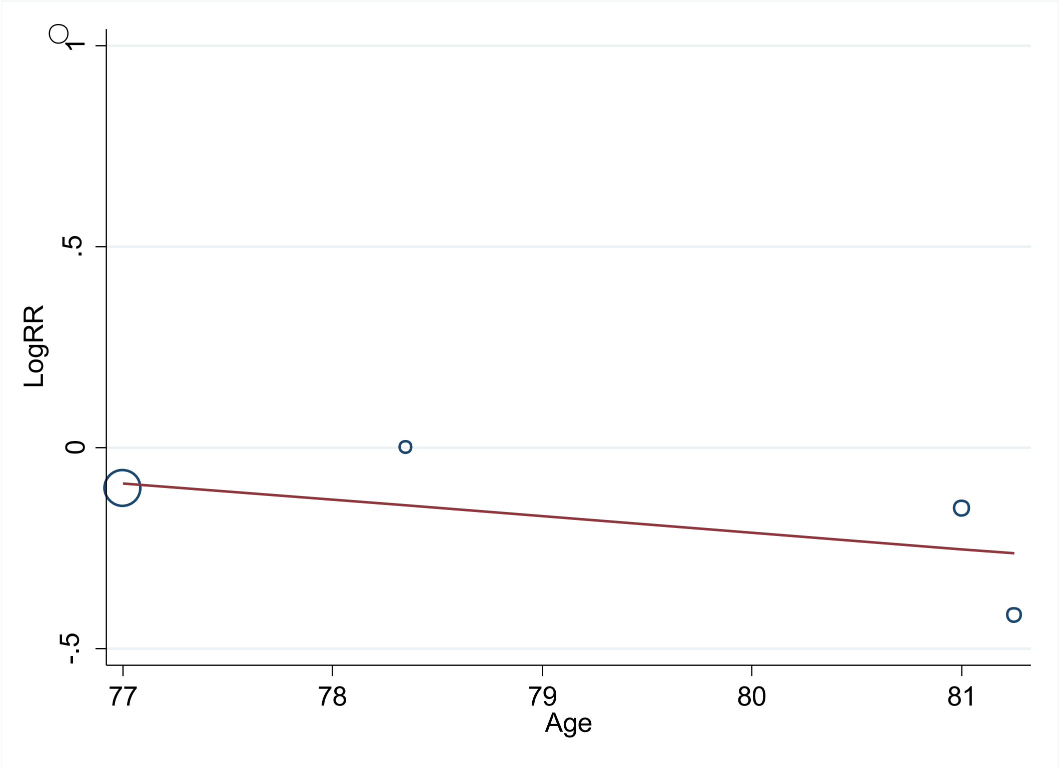

Figure S7 Meta-regression representative bubble plot for mean age

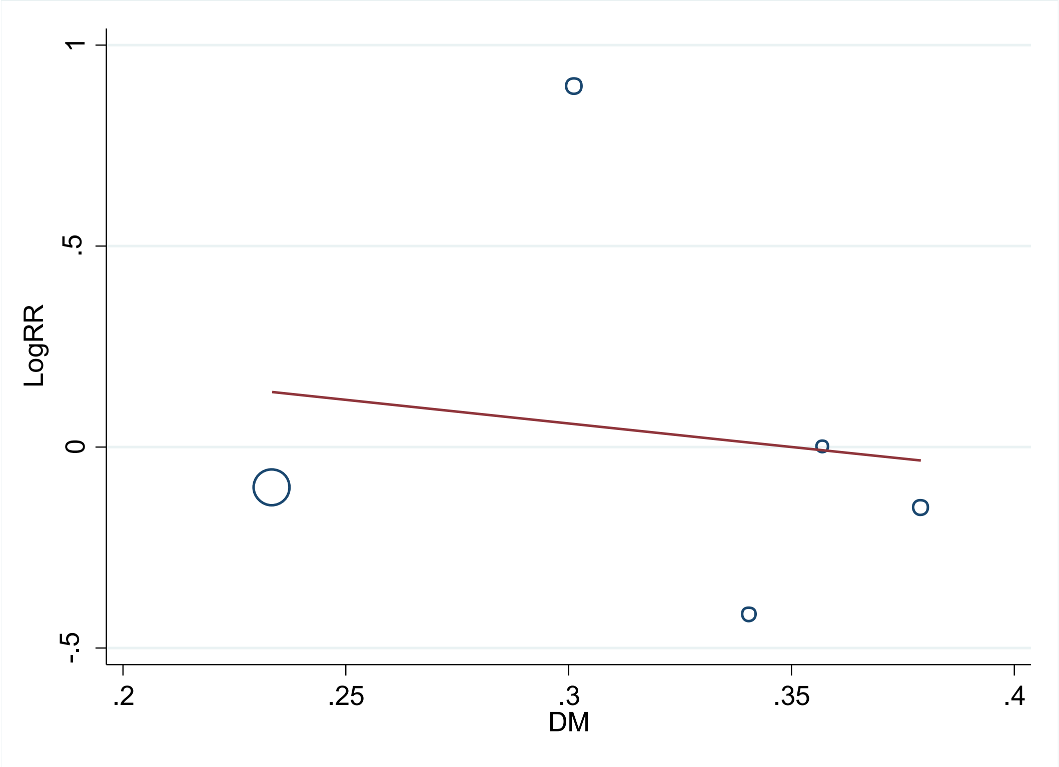

Figure S8 Meta-regression representative bubble plot for diabetes mellitus

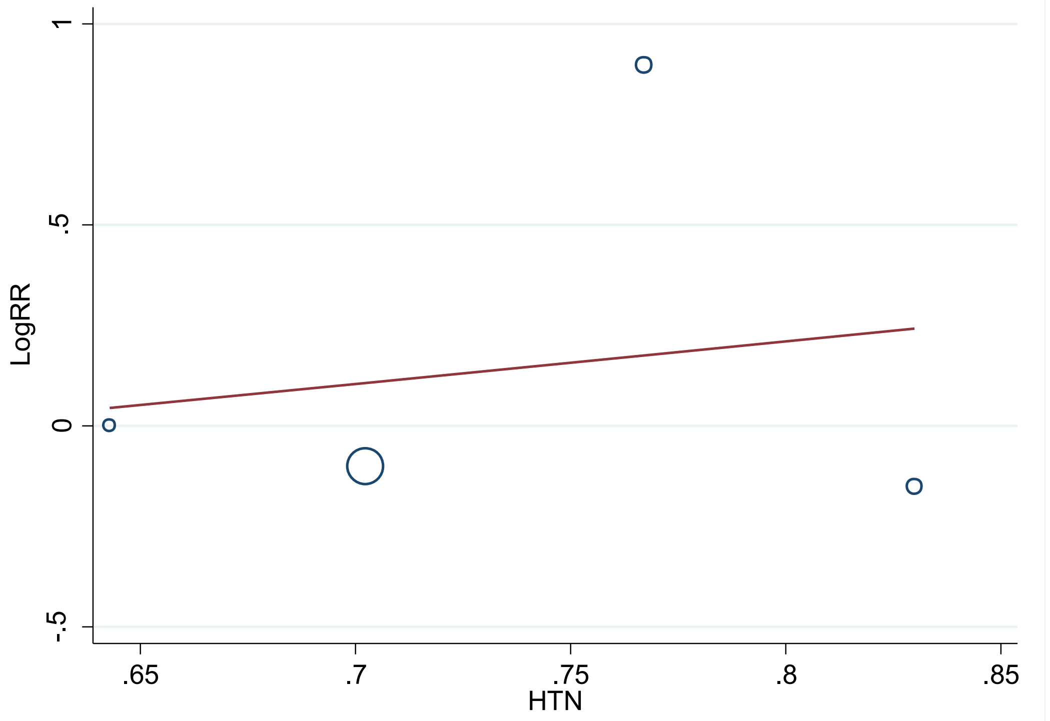

Figure S9 Meta-regression representative bubble plot for hypertension

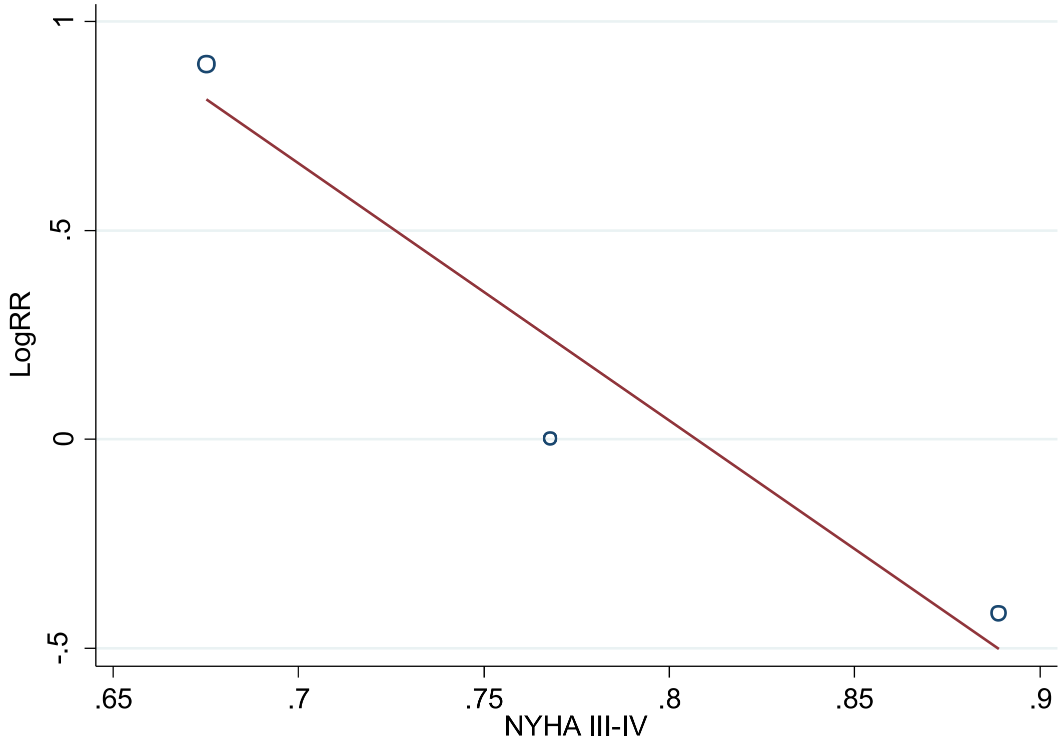

Figure S10 Meta-regression representative bubble plot for heart failure with NYHA III-IV symptoms

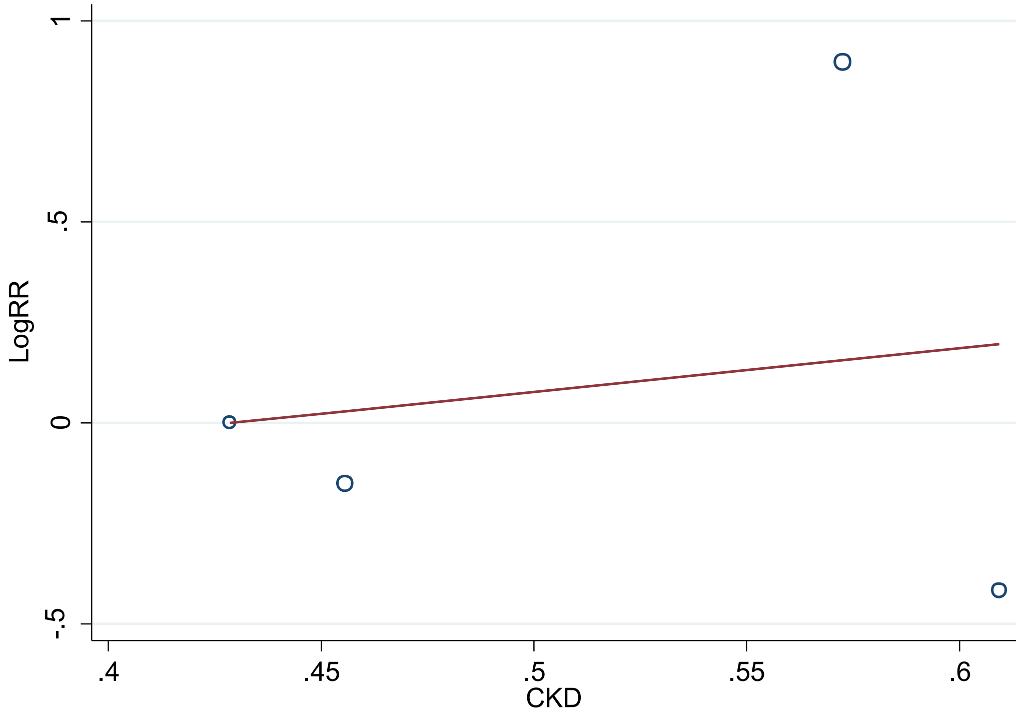

Figure S11 Meta-regression representative bubble plot for chronic kidney disease (GFR <60 ml/min/1.73 m^2)

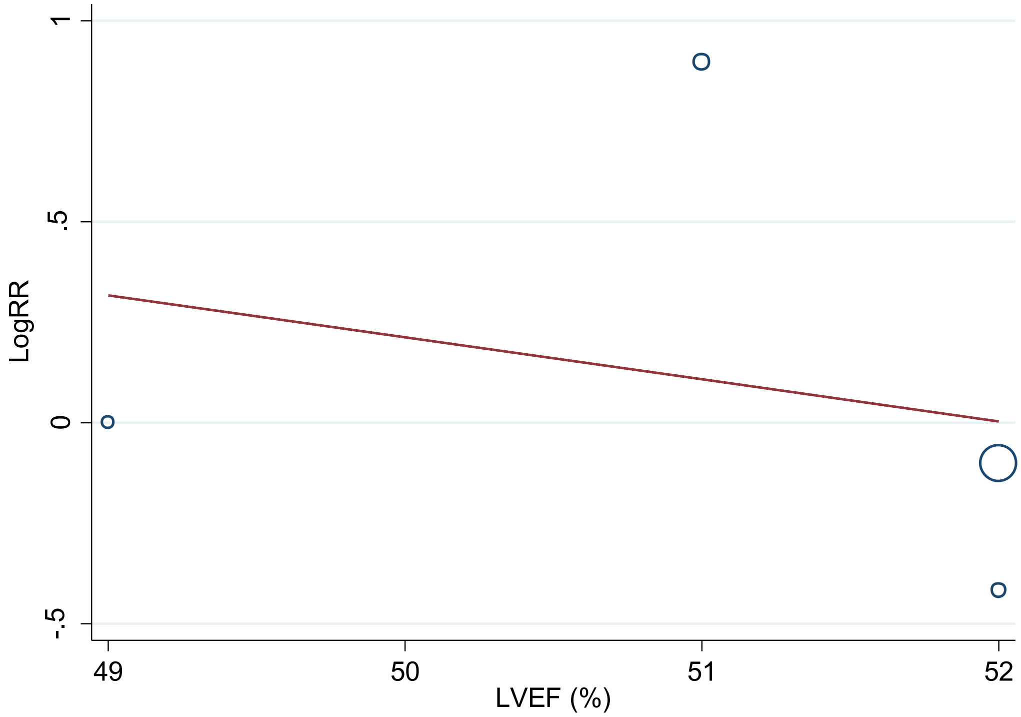

Figure S12 Meta-regression representative bubble plot for mean left ventricle ejection fraction (LVEF, %)

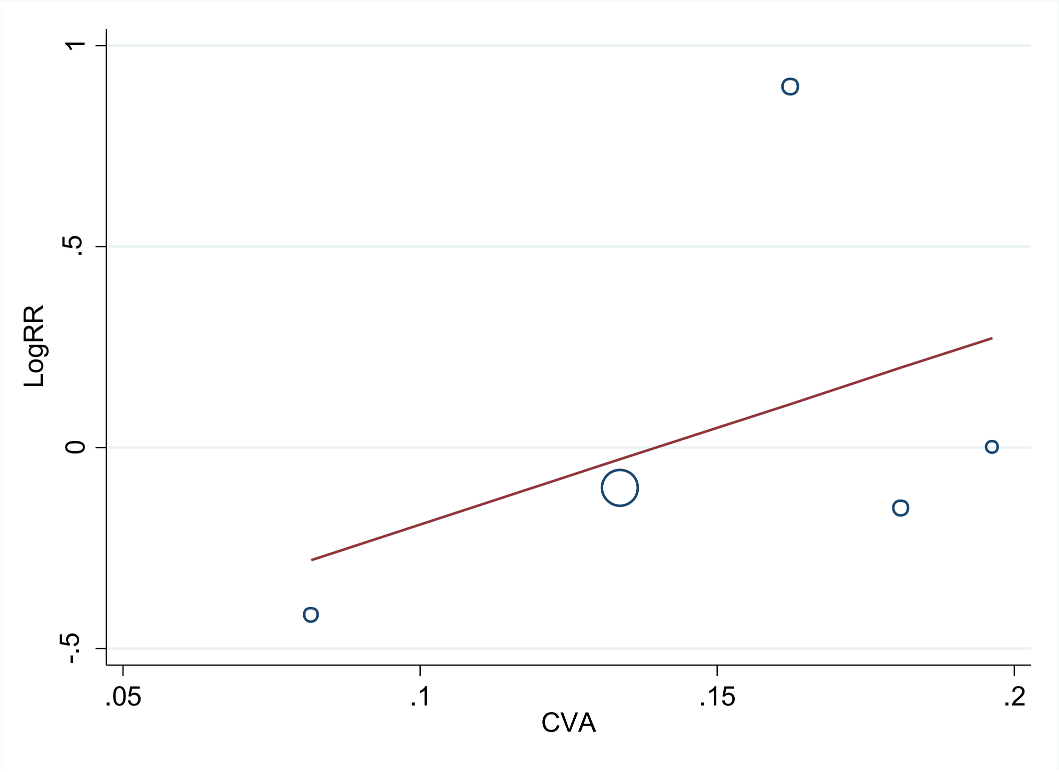

Figure S13 Meta-regression representative bubble plot for previous cerebrovascular accident (CVA)

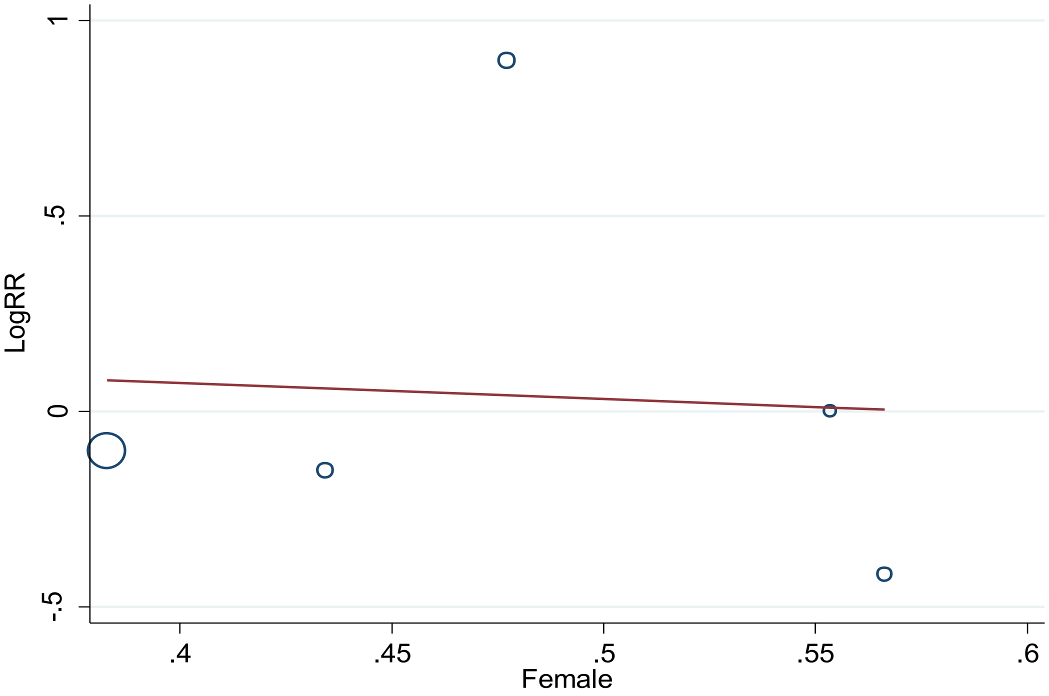

Figure S14 Meta-regression representative bubble plot for female gender

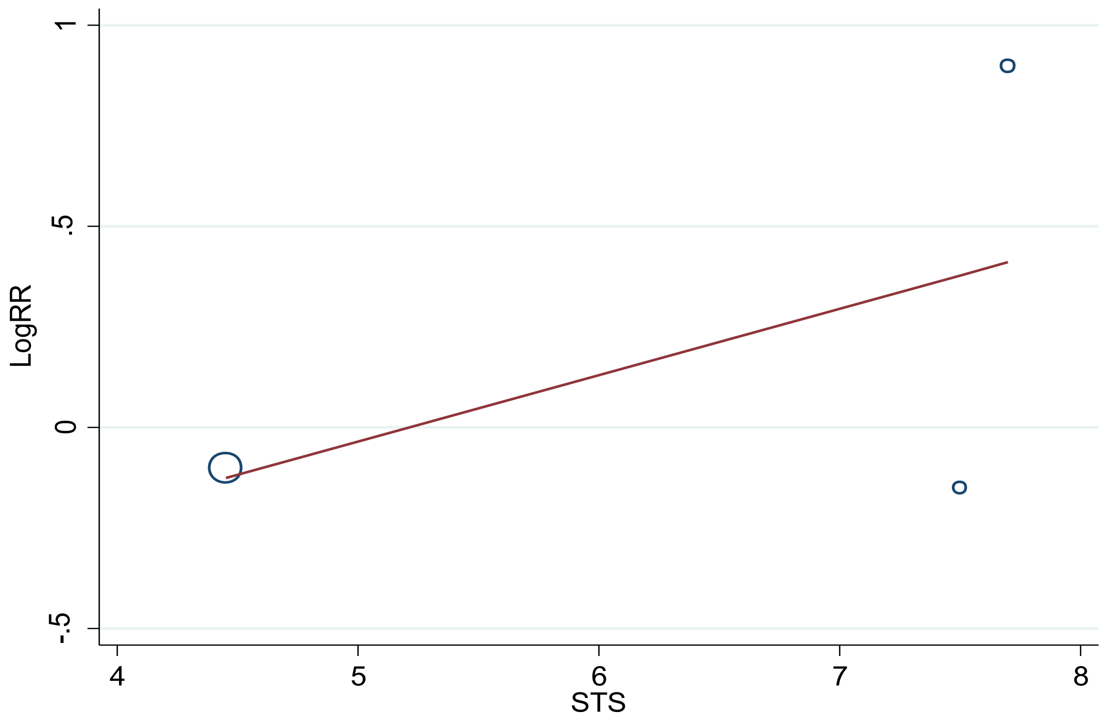

Figure S15 Meta-regression representative bubble plot for STS score

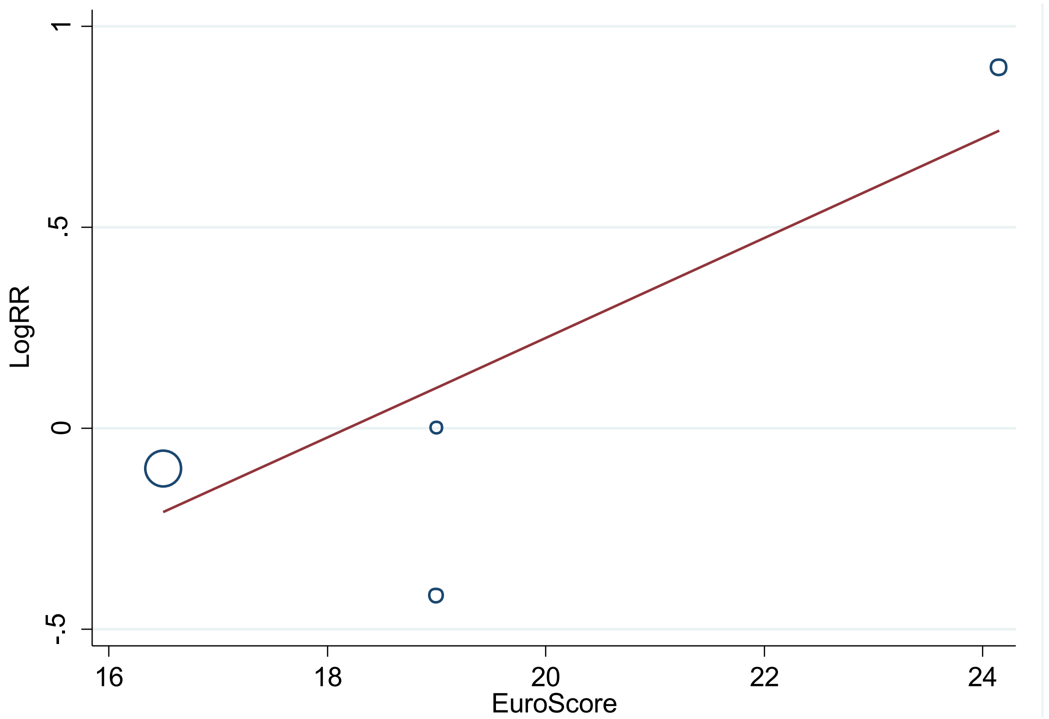

Figure S16 Meta-regression representative bubble plot for EuroScore

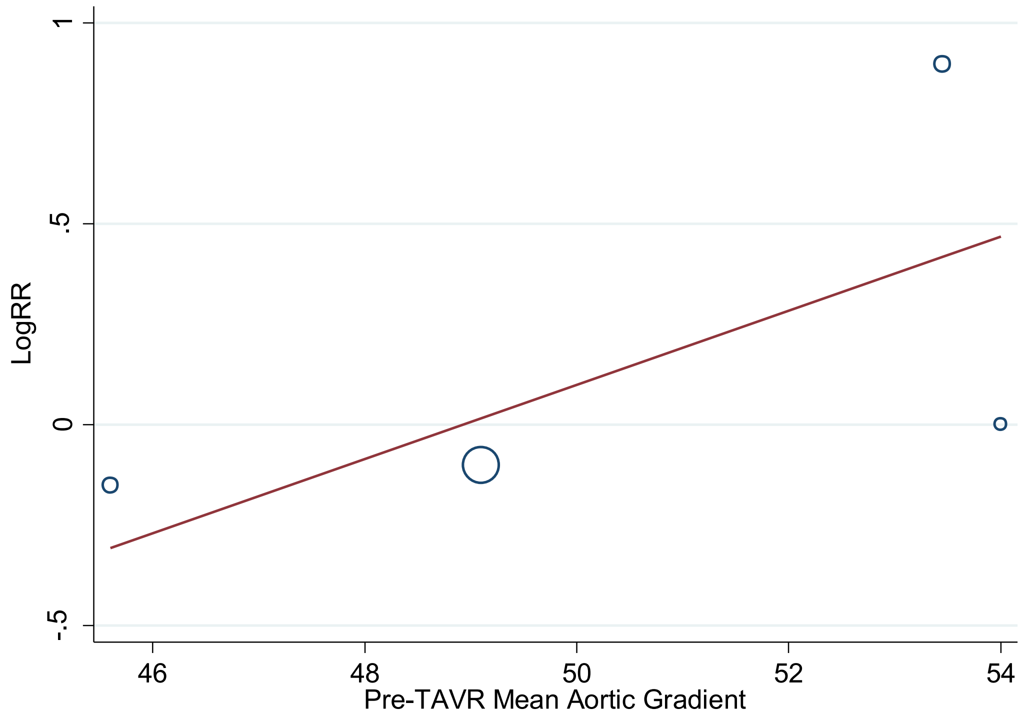

Figure S17 Meta-regression representative bubble plot for pre-TAVR Mean Aortic Gradient

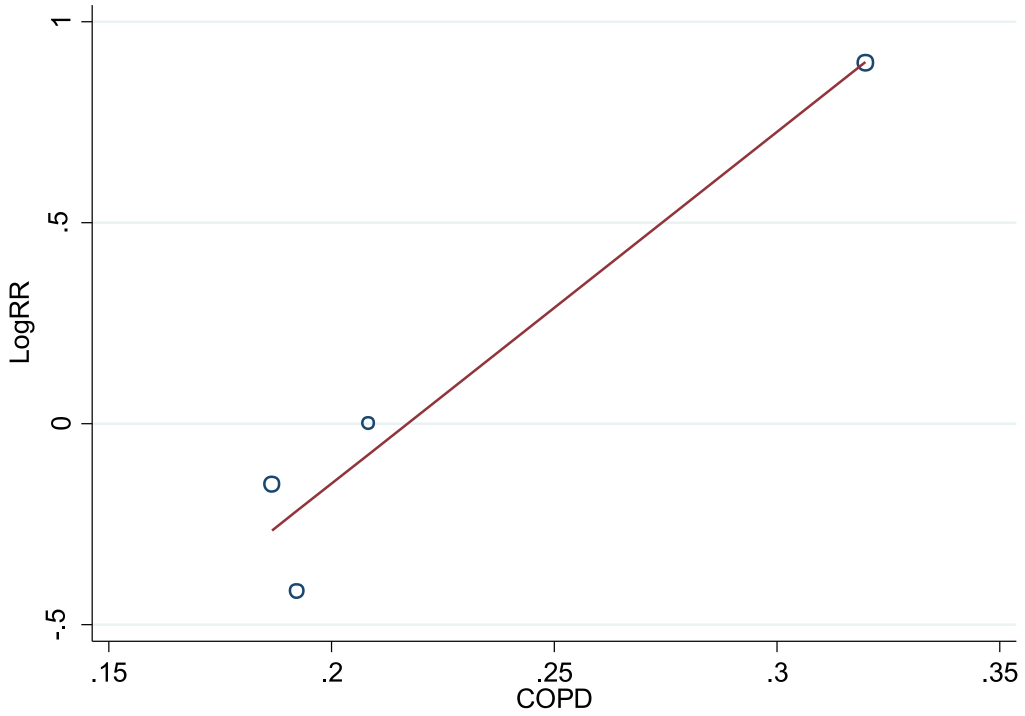

Figure S18 Meta-regression representative bubble plot for Chronic Obstructive Pulmonary Disease (COPD)

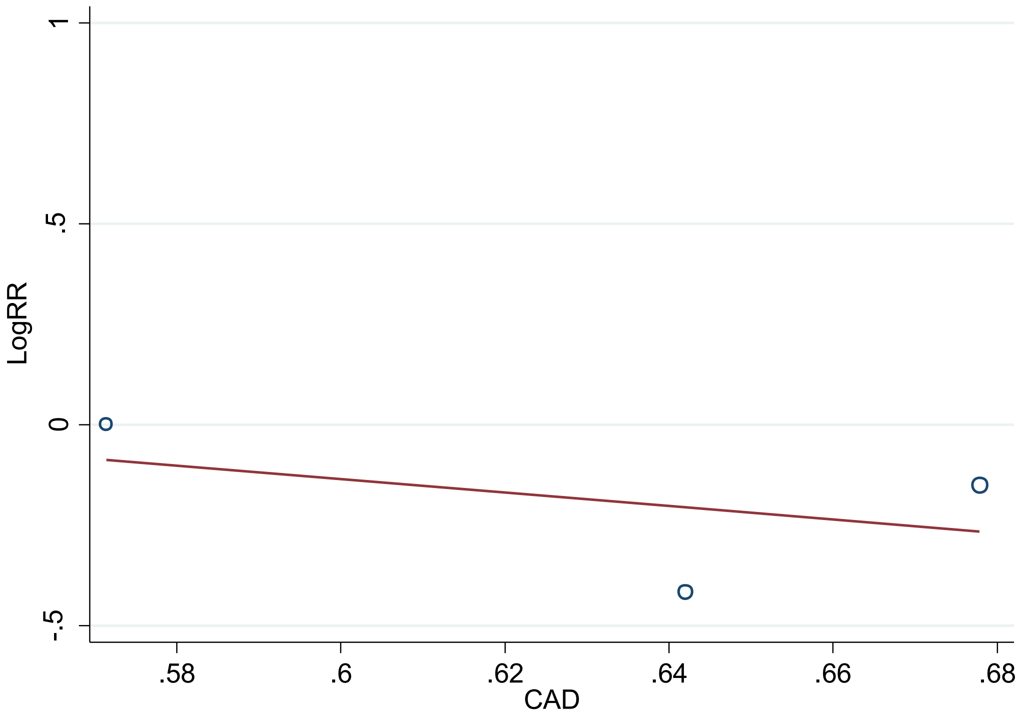

Figure S19 Meta-regression representative bubble plot for Coronary Artery Disease (CAD)

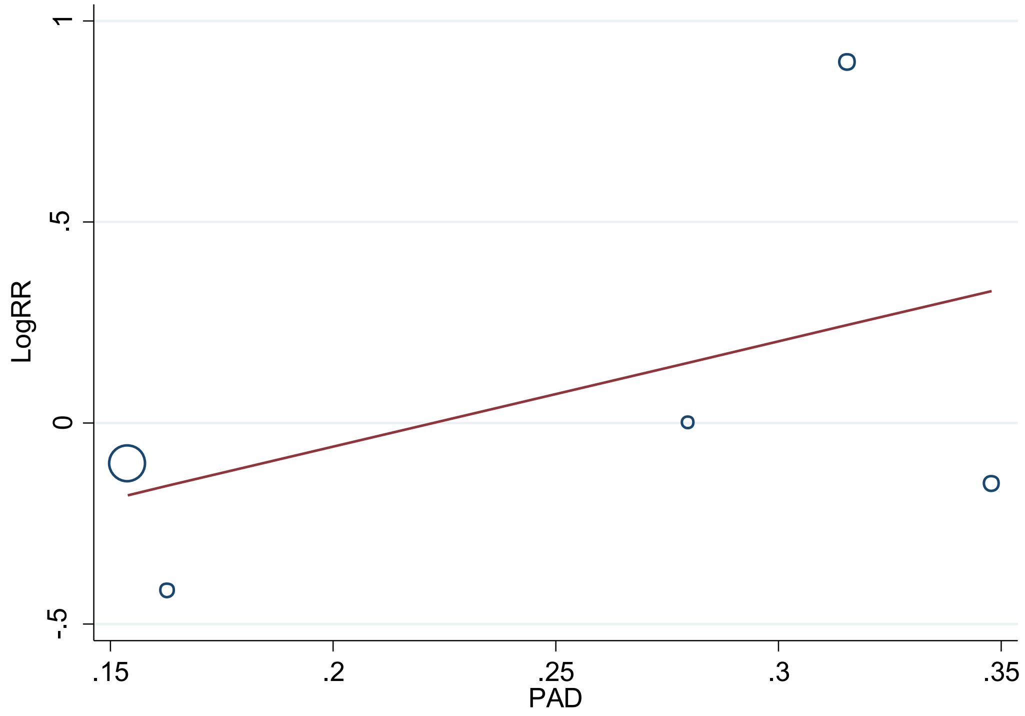

Figure S20 Meta-regression representative bubble plot for Peripheral Artery Disease (PAD)

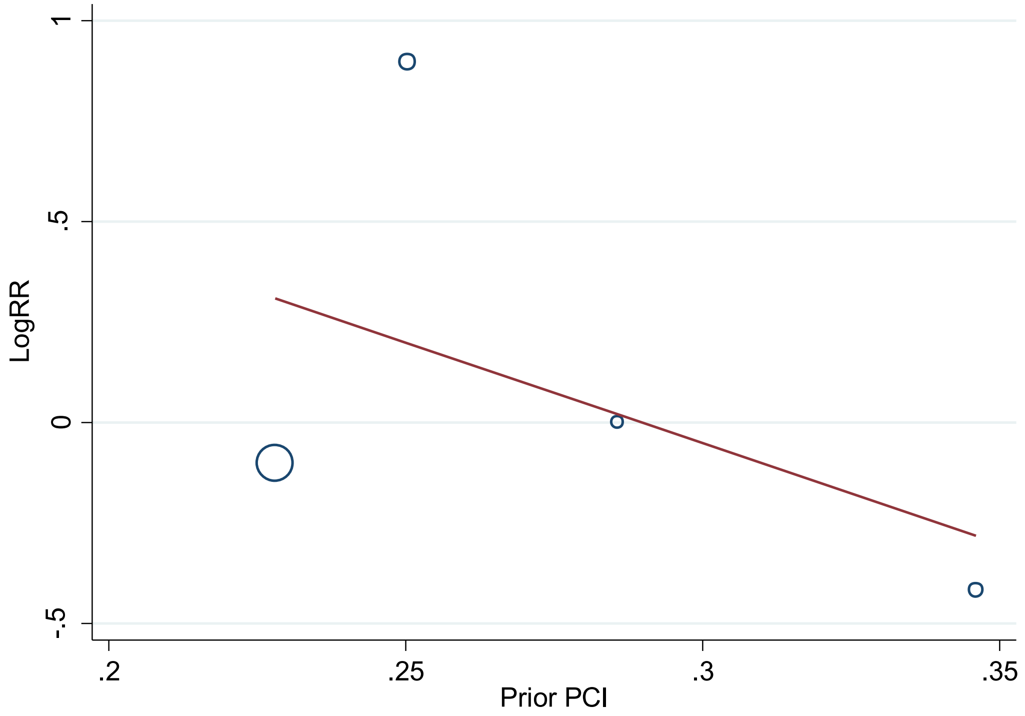

Figure S21 Meta-regression representative bubble plot for prior Percutaneous Coronary Intervention (PCI)

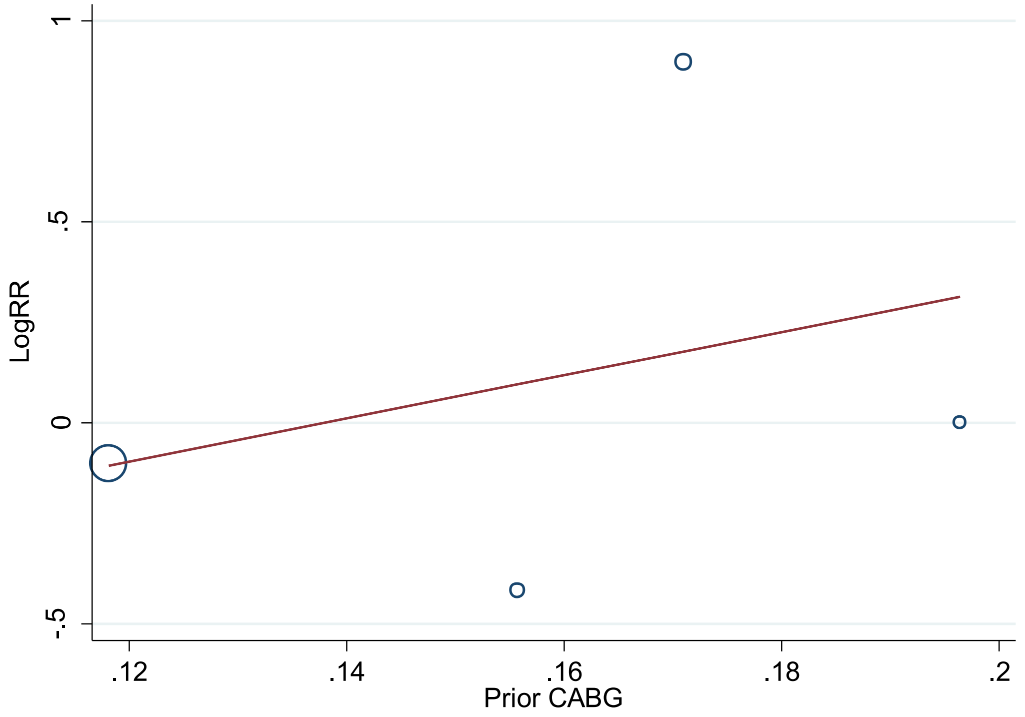

Figure S22 Meta-regression representative bubble plot for prior Coronary Artery Bypass Graft (CABG)
